# Incorporating equity in infectious disease modeling: Case study of a distributional impact framework for measles transmission

**DOI:** 10.1016/j.vaccine.2021.03.023

**Published:** 2021-05-18

**Authors:** Tigist Ferede Menkir, Abdulrahman Jbaily, Stéphane Verguet

**Affiliations:** a Center for Communicable Disease Dynamics, Department of Epidemiology, Harvard T.H. Chan School of Public Health, Harvard University, Boston, MA, USA; b Department of Global Health and Population, Harvard T.H. Chan School of Public Health, Harvard University, Boston, MA, USA

**Keywords:** Dynamic transmission modeling, Measles, Vaccination, Equity, Socioeconomic status, Social contact matrices

## Abstract

**Introduction:**

Deterministic compartmental models of infectious diseases like measles typically reflect *biological* heterogeneities in the risk of infection and severity to characterize transmission dynamics. Given the known association of socioeconomic status and increased vulnerability to infection and mortality, it is also critical that such models further incorporate *social* heterogeneities.

**Methods:**

Here, we aimed to explore the influence of integrating income-associated differences in parameters of traditional dynamic transmission models. We developed a measles SIR model, in which the Susceptible, Infected and Recovered classes were stratified by income quintile, with income-specific transmission rates, disease-induced mortality rates, and vaccination coverage levels. We further provided a stylized illustration with secondary data from Ethiopia, where we examined various scenarios demonstrating differences in transmission patterns by income and in distributional vaccination coverage, and quantified impacts on disparities in measles mortality.

**Results:**

The income-stratified SIR model exhibited similar dynamics to that of the traditional SIR model, with amplified outbreak peaks and measles mortality among the poorest income group. All vaccination coverage strategies were found to substantially curb the overall number of measles deaths, yet most considerably for the poorest, with select strategies yielding clear reductions in measles mortality disparities.

**Discussion:**

The incorporation of income-specific differences can reveal distinct outbreak patterns across income groups and important differences in the subsequent effects of preventative interventions like vaccination. Our case study highlights the need to extend traditional modeling frameworks (e.g. SIR models) to be stratified by socioeconomic factors like income and to consider ensuing income-associated differences in disease-related morbidity and mortality. In so doing, we build on existing tools and characterize ongoing challenges in achieving health equity.

## 1 Introduction

Dynamic compartmental models aimed at capturing transmission of infectious pathogens have been largely used to describe and anticipate the propagation of infectious diseases in populations worldwide [1]. Often, to better represent real-world differences in disease dynamics across different population subgroups, extensions to the simplest transmission models (e.g. Susceptible-Infected-Recovered or SIR models) usually lift the assumption of homogenous mixing and susceptibility to disease, and explicitly account for heterogeneities in transmission across age groups and distinct “risk groups” or for differential health-promoting behaviors in response to prevailing rates of disease [2], [3], [4], [5]. One important driver of glaring disparities in the risk of acquiring disease that is often underexplored in these approaches is social determinants of health, in particular socioeconomic group partitions, beyond binary risk definitions of high- and low-risk “social groups” [6]. Certain socioeconomic groups, notably the poorest, can be more vulnerable to infectious diseases due to increased contact rates (potentially associated with overcrowding) and to increased susceptibility to disease (e.g. with undernutrition) [7]. Therefore, incorporation of socioeconomic status in disease transmission models is critical, especially for diseases like measles where transmission rates can scale with population size such that the risk of infection could be greatly amplified in lower socioeconomic groups with often larger household sizes [8].

Economic evaluation approaches, such as cost-effectiveness analyses, often build on dynamic modeling exercises to assess the value for money of disease control interventions [9], [10]. Such analyses can appraise the direct and indirect costs for individuals and governments associated with the implementation of a given intervention, and compare them against the benefits gained by the intervention, notably averted disease-related cases and deaths per budget expenditure [9]. Various perspectives can guide the designation of costs and benefits in such exercises. For instance, an emphasis can be placed on individuals and households, especially the most economically disadvantaged, by comparing intervention impact across socioeconomic groups [11], [12], [36]. This can highlight the interventions which most benefit lower income populations and ultimately work to enhance equity [11], [12], [36].

However, with the exception of a few studies [3], [6], most analyses focus on the issue of equity and distributional outcomes using static approaches or “ex-post facto” (see for example Chang et al. [13]). That is, dynamic transmission models do not usually consider heterogeneities in infection and progression to disease across different income groups. For example, the aggregate outputs of dynamic models can be stratified to estimate case counts across income groups, in lieu of directly modeling the underlying heterogeneities in disease transmission. Some economic evaluations may also choose to place a greater emphasis on examining the financial consequences of illness to the poor (e.g. magnitude of disease-related out-of-pocket costs and induced impoverishment) [12], [14]. In contrast, transmission models detail disease propagation in a population, which requires a prime consideration of heterogeneities in transmission and survival, and how these shape the evolution of infection and mortality over time. Overlooking variations in the transmission process across income groups may result in failing to capture the full extent of disease burden in the poorest groups, and correspondingly the comprehensive benefits of a given intervention (e.g. vaccination) among these groups.

In this paper, we propose an income-stratified SIR model that explicitly accounts for income heterogeneities in risk of infection and mortality, through varying disease-related parameters, notably transmission rates and disease-induced mortality rates. The model allows for the quantification of the relative impacts, across income quintiles, of varying measles vaccine coverage assumptions. To showcase its properties and implications, we provide a stylized illustration for an Ethiopian setting. Ethiopia is a low-income country with the second largest population in Africa [15] and still grapples with a high measles burden [16], [17]. The country is regularly confronted with outbreaks [18] fueled in part by low levels of vaccination coverage (59% nationally) and considerable disparities by socioeconomic status: the lowest and highest wealth quintiles reported coverage rates of 42% and 83%, respectively, in 2019 [19].

## 2 Methods

We detail our approach in the following subsections. First, we present our income-stratified SIR model and a selection of input parameters. Second, we consider five key vaccination cases for which we quantified the consequences on disparities in measles mortality.

### 2.1 Model description

We developed a stylized deterministic compartmental model of measles transmission stratified by income quintile. Note that, while age clearly plays a role in modulating measles transmission, evidence for heterogeneities by socioeconomic status (SES) in the risk of measles infection has also been established, which further motivates our analysis [20]. That is, here, we primarily aim to illustrate the effects of income-associated differences in key parameters shaping transmission.^1^

Our model consisted of three compartments: Susceptible (S), Infected (I), and Recovered (R) classes, for each income quintile, with our main input parameters being the quintile-specific values for disease transmission rates, measles case-fatality ratios (CFRs), and vaccination coverage. Specifically, the stylized SIR model was recast as:

(1) $$\begin{array}{c} \frac{dS_{i}}{\mathrm{dt}}=\left(1-{\mathrm{ep}}_{i}\right)\upsilon_{i}N_{i}-\sum_{j}{\beta_{\mathrm{ij}}S}_{i}I_{j}-\mu_{i}S_{i},\\ \frac{dI_{i}}{\mathrm{dt}}=\sum_{j}{\beta_{\mathrm{ij}}S}_{i}I_{j}-{(\mu }_{i}+\gamma )I_{i},\\ \frac{dR_{i}}{\mathrm{dt}}={\mathrm{ep}}_{i}\upsilon_{i}N_{i}+\left(1-d_{i}\right)\gamma I_{i}-\mu_{i}R_{i},\\ \frac{dD_{i}}{\mathrm{dt}}=d_{i}\gamma I_{i} \end{array}$$

with the initial conditions (at t=0): $S_{i}\left(0\right)=\left(1-ep_{i}\right)N_{i,0}$ and $R_{i}\left(0\right)=ep_{i}N_{i,0}$. The compartment *D* tracks measles deaths. For quintile *i*, $p_{i}$ is vaccination coverage, $N_{i,0}$ is the number of individuals (initially at t=0), $\mu_{i}$ is background mortality rate, and *d_i_* is the measles CFR. $\beta_{\mathrm{ij}}$ is the transmission rate from quintile *j* to quintile *i*, which is multiplied by the number of infected in quintile *j* (*I_j_*), and summed over all quintiles *j* to yield the force of infection per susceptible in quintile *i* (*S_i_*). By design (model equations (1)), density-depending mixing is assumed, where contact rates grow with population size [2]. For ease of interpretation, we standardized our compartment sizes using the same initial sizes ($N_{i,0}=N_{0}$) per quintile. Lastly, we assumed a constant recovery rate γ across quintiles and vaccine efficacy e.

### 2.2 Model parametrization

The model was parameterized using both published modeling parameters and secondary data from Ethiopia (see details in the supplementary webappendix section 1) [22], [23], [24].

While previous work has simulated contact matrices for Ethiopia across age groups and by rural/urban residence [25], [26], to our knowledge, neither empirical nor simulated contact matrix data across income groups are available for low- and middle-income country (LMIC) settings. Therefore, we elaborated five possible types of income-stratified transmission matrices (scenarios 1–5). Each transmission matrix reports the “effective contact rate” between a susceptible and an infected individual across any two quintiles [27].

**Fig. 1:**
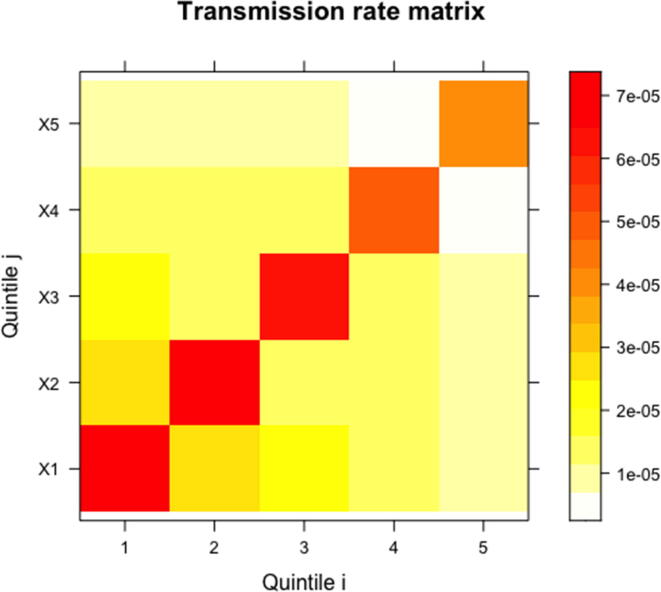
Sample transmission matrix from scenario 1 simulations. Diagonal and off-diagonal cells indicate the within- and cross-quintile transmission rate terms (i.e. mean rate of infections per day between any two susceptible and infected individuals from quintiles i and j), respectively. Darker colors indicate greater transmission rates.

For the elaborated scenarios 1, 2, and 3, we simulated transmission matrices with varying assumptions on how the quintile-specific reproductive number (R_0,i_ for quintile *i*) attributed partitioned (i.e. a certain fraction within [0;1]) to within- and cross-quintile transmission [5]. We first sampled five values for the overall R_0,i_'s ($R_{0,i}=R_{\mathrm{ii}}+\sum_{j=1(j\neq i)}^{5}R_{\mathrm{ij}}$) portioning each of these in descending order to yield the corresponding R_ii_ (diagonal term (i,i) of the matrix) and remaining $\sum_{j=1(j\neq i)}^{5}R_{\mathrm{ij}}$ (sum of off-diagonal terms in row i of the matrix). For example, a sample of R_0,i_'s might consist of the following five values: 10.2; 11.1; 12.4; 14.4; and 17.9, where the largest value (17.9) would be assigned to the lowest quintile (i.e. R_0,1_ = 17.9).^2^ We then sequentially sampled the cross-quintile R_ij_ (j≠i) terms per quintile i, with the constraint that these values sum to the apportioned sum of quintile i’s cross-quintile terms (i.e. $\sum_{j\neq i}^{5}R_{\mathrm{ij}})$. For our base-case scenario 1, we assumed that within- and cross-quintile transmission each constituted half of total R_0,i_. For simplicity, the resulting transmission matrix was assumed to be symmetrical, with $\beta_{\mathrm{ij}}=\beta_{\mathrm{ji}}$ (Fig. 1). Full detail of the algorithm implemented is given in webappendix (section 2).

For scenario 4, we broadly interpreted SES contact data from a different setting, Mexico, which used information on individual-level expense histories documented at banks as well as phone call and text data to classify individuals into income deciles and impute contact rates by decile [28]. In these data, the contact matrices reported the ratio of the “number of links” (phone calls or texts) observed between individuals in all possible decile pairings and the number of links that would be expected in a social network with random mixing [28] (see scenario 4, webappendix). Lastly, scenario 5 assumed homogeneous mixing (i.e. constant R_0_ across all quintiles).

### 2.3 Cases of distributional vaccination

We studied the influence of distributional vaccination (first dose of measles vaccine, i.e. MCV1) using five illustrative cases: (i) status quo coverage rates (i.e. DHS quintile-specific MCV1 coverage); (ii) flat coverage (equal to DHS mean MCV1 coverage); (iii) 50% relative increase (from status quo (i)) in coverage in each quintile; (iv) coverage in each quintile set to equal coverage of top quintile; and (v) full (100%) coverage. For each quintile, we computed the difference in the risk of measles mortality under each vaccination case (ii, iii, iv, and v) compared to the base-case (i) and accounted for the total final population size in each quintile to yield the associated reduction in disparities in measles deaths across quintiles. To formally assess these disparities, we computed concentration indeces (CIs) (as described in the webappendix, section 3) [29].

The model was run for 400 days, repeated over *n* = 1000 iterates to account for uncertainty in all input parameters, and we reported point estimates and 95% uncertainty intervals (UIs) across simulations. All input parameters (and corresponding distributions) are listed in Table 1 (with further details provided in the webappendix). The analysis was run using R statistical software (version 3.6.1) [30]. All code is publically available on Github (https://github.com/goshgondar2018/equity_SIR).

**Table 1:** Values, probability distributions, descriptions, and sources of all input parameters used in the distributional Susceptible-Infected-Recovered (SIR) model.

Parameter	Definition	Value	Probability distribution	Unit	Source(s)
*p_1_,…, p_5_*	Vaccination coverage (%); income quintiles 1–5	43; 50; 54; 59; 74	Triangular(0.5*Value, 1.5*Value)	Dimensionless	[22]
$\upsilon_{1}$*, …,*$\upsilon_{5}$	Birth rate (per 1000); quintiles 1–5	43; 37; 33; 29; 17	Uniform(0.5*Value, 1.5*Value)	1000^-1^ yr^−1^	[22], authors’ assumption
*f*	Probability of successful infection	0.03	N/A	Dimensionless	Authors’ assumption based on [23]
$R_{0}$	Population-wide reproduction number	16	Uniform(10,22)	Dimensionless	Authors’ assumption based on [23]
$\mu_{1}$*, …,*$\mu_{5}$	Crude death rate (per 1000); quintiles 1–5	Set to: 43; 37; 33; 29; 17	Uniform(0.5*Value, 1.5*Value)	1000^-1^ yr^−1^	Authors’ assumption
*d_1, …,_ d_5_*	Case-fatality ratio (%); quintiles 1–5	2.18; 1.89; 1.60; 1.31; 1.02	Uniform(0.5*Value, 1.5*Value)	Dimensionless	Authors’ assumption derived from [22], [24]
γ	Recovery rate	0.0714	Inv-Gamma(shape = 15,1)	day^−1^	[23]
e	Vaccine efficacy	0.85	N/A	Dimensionless	[21]

Quintile 1 = poorest; quintile 5 = richest.

## 3 Results

**Fig. 2:**
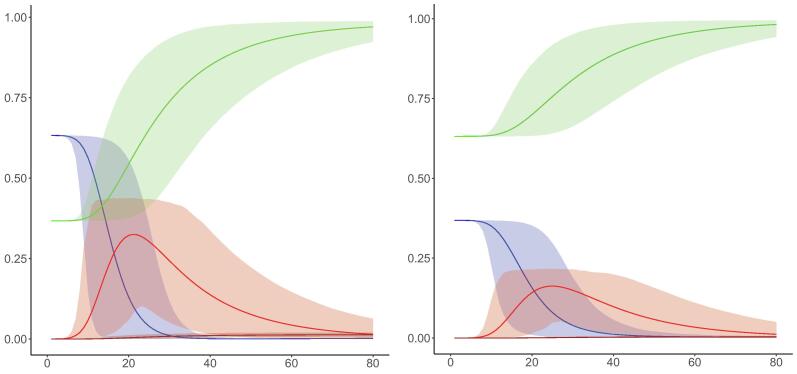
Susceptible (blue), Infected (red), Recovered (green), and Deceased (brown) model dynamics for quintile 1 (left) and 5 (right) under transmission scenario 1. Solid lines indicate mean values while shaded areas indicate 95% uncertainty intervals for each compartment.

We first report on the dynamic evolution of the S, I, R and D proportions for each quintile under scenario 1 (Fig. 2; webappendix Fig. S1). Overall, we observe behaviors consistent with traditional SIR models: a steep decline in the S population shortly after epidemic onset, followed by a rapid increase in the I population, and an increase and eventual plateauing of the R population. We see important differences across quintiles: the lowest quintile reports a peak in I that noticeably exceeds that of the highest quintile (0.33 vs. 0.16), with its D population reaching a higher level at the conclusion of the study period (0.014 vs. 0.004), indicating a higher risk of infection at the epidemic’s apex and a higher risk of death among those in the lowest quintile. The R population for quintile 5 stabilizes at a higher level than for quintile 1, largely explained by relatively higher vaccination coverage in quintile 5. The proportion of measles deaths decreases sharply with income, with the most perceptible differences between the bottom four quintiles and the top quintile (Fig. S1).

Second, expectedly, we observe slightly varying infected trajectories under each transmission scenario (1–5; Fig. S2). While the half-, high- and low- within-quintile transmission scenarios (1, 2, 3) broadly overlap, the low within-quintile transmission scenario yields a lower infected peak, followed by the half and high scenarios. That is, with mixing more concentrated within quintiles, the early surge in infections becomes more pronounced, again testifying the clear gradient in reduced risk with increasing quintile described previously. The homogeneous mixing scenario (5) presents the lowest peak infection in all quintiles. Lastly, while the Mexico contact survey scenario (4) suggests slightly earlier dynamics among each quintile, due to relatively more intensified transmission rates imputed, this scenario still replicates the sharp decline in risk of infection with increasing quintile observed in the other scenarios.

**Fig. 3:**
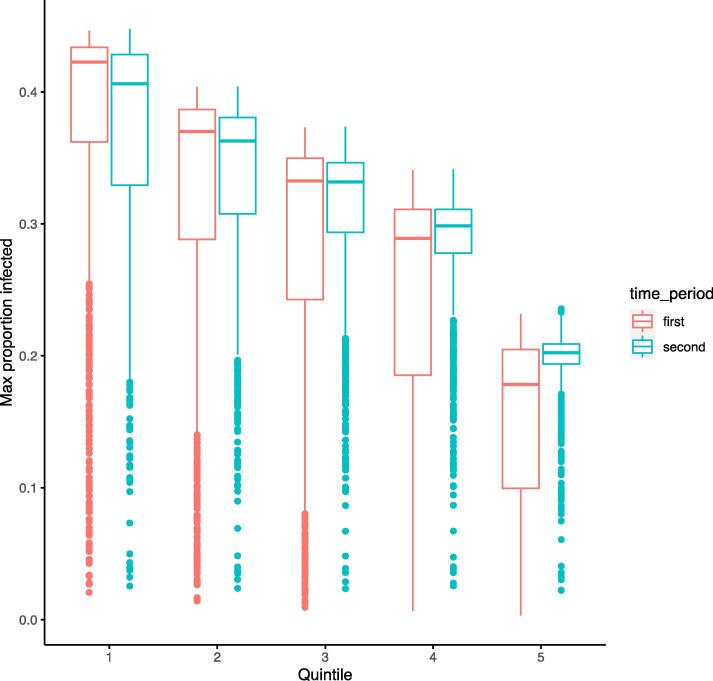
Distribution of the maximum proportion of individuals who are infected in the first 20 days of the outbreak (pink) vs. 21–40 days into the outbreak (blue), by quintile (1 = poorest; 5 = richest), under transmission scenario 1.

When further examining scenario 1 (Fig. 3), we find notable variations in the distribution of the I population over time in each quintile (e.g. during first twenty days vs. subsequent period, when peak proportions are reached for all infected classes). This stresses the marked gradient in the distribution in the proportion of infected individuals over time, most acutely at the onset. During the first twenty days, the lower quintiles reach higher maximum infection proportions than the upper quintiles (e.g. median of 0.42 vs. 0.18 for the lowest and highest quintiles, respectively), signaling that a majority of infections occur most explosively for the lowest quintiles within the early period of the outbreak.

**Fig. 4:**
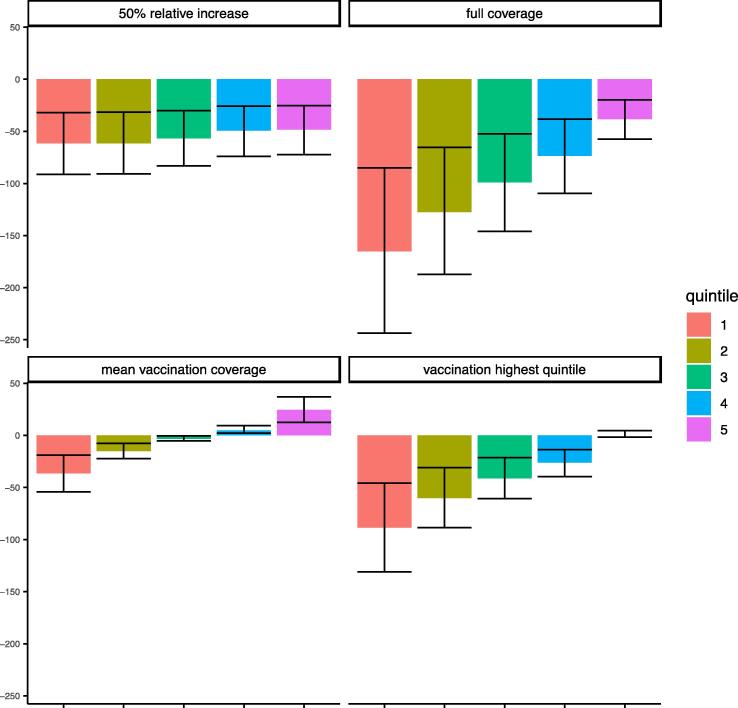
Reduction in the number of measles deaths for each quintile (among 15,000 individuals per quintile) under each vaccination strategy (ii; iii; iv; v) compared to the base-case strategy (i) (means and 95% uncertainty intervals are reported). (i) = status quo coverage rates (i.e. DHS quintile-specific coverage); (ii) = flat coverage (equal to DHS mean coverage); (iii) = 50% relative increase (from status quo (i)) in coverage in each quintile; (iv) = coverage in each quintile set to equal coverage of highest quintile; (v) = full (100%) coverage.

With respect to distributional vaccination (Fig. 4), increasing coverage rates (*p_j_* across quintiles) appears to yield, as anticipated, reduced disease mortality, particularly for lower quintiles. The full (100%) coverage case shows the greatest reduction in deaths among lower quintiles: 165 (95% UI: 85–244) and 128 (65–187) averted deaths in quintiles 1 and 2, respectively, and only 38 (20–58) averted deaths in quintile 5. Quintiles 1 and 2 also see marked mortality reductions under flat vaccination coverage corresponding to the top quintile’s coverage level. When coverage is equalized across all quintiles to the mean coverage rate, quintiles 4 and 5 actually see an increase in mortality, due to reduced coverage rates in those quintiles relative to their baseline level (i). In nearly all cases, quintile 5 would benefit from increased vaccination in all other quintiles: this can be attributed to both increased protection from infection within the quintile and the decline in cross-quintile transmission that results from reducing infections in other quintiles with whom transmission rates are non-trivial. When vaccination coverage is increased by 50% solely for quintile 5, the deaths averted in this quintile actually fall just short of the deaths averted when vaccination coverage is increased by 50% in all quintiles.

All five vaccination settings translate to differential measles mortality disparities (under transmission scenario 1). Under the base-case (i), the CI was estimated at −0.22 (95% UI: −0.35 to −0.08), indicating that the lowest quintiles are disproportionately affected by measles mortality. The remaining vaccination cases all indicate reduced disparities, with the exception of the 50% relative increase case, with a CI at −0.35 (−0.46 to −0.22), a consequence of the marked additional benefits accrued in the top quintiles with relatively modest changes in the bottom quintiles. The flat vaccination case at the mean coverage level is associated with the greatest inequality reduction, reporting a CI of −0.15 (−0.28 to 0.00), closely followed by setting coverage levels to that of the highest quintile (similar CI). Despite significant mortality reductions resulting from the full (100%) coverage case, the corresponding CI is more negative (−0.18; −0.31 to −0.03), implying marginally lower equity gains.

## 4 Discussion

In this paper, we attempted to explicitly incorporate income-associated heterogeneities in social vulnerability to infection and disease-induced mortality, and we showed, via a stylized illustration of measles, how these considerations might alter the distribution of infectious disease burden. Such modeling is critical for anticipating infection and mortality risk profiles across socioeconomic groups and prioritizing vaccination to the populations who would most benefit. We presented here a simple example of how SIR models for infectious diseases like measles, which are intimately tied to social determinants, could structurally account for income.

While our income-stratified model yielded dynamics similar in nature to that of the standard (i.e. non income-stratified) SIR model, we could also observe pronounced differences in the scale of these dynamics across income quintiles. That is, with higher within-quintile transmission, for instance, the bottom quintiles see taller peaks in the size of their infected populations and generally more explosive increases in infections, and greater disease-induced mortality, with a proportionate decrease in these patterns with increasing income.

Furthermore, marginal increases in vaccination coverage could result in significant gains, with the greatest accrual of benefits in the poorest. Increasing vaccination to full coverage and leveling all quintiles’ coverage rates to that of the highest quintile would be associated with a striking reduction in deaths for all quintiles, and most acutely for the poorest. Notably, both increasing coverage for all quintiles to that of the highest quintile, or setting coverage rates all equal to mean vaccination coverage, would be associated with drops in measles mortality disparities. Lastly, it is important to note that the wealthiest quintiles would benefit from enhanced vaccination for all quintiles, and thus would also derive benefits from public health preventative strategies aimed at alleviating already existing inequalities among the poorest.

However, we acknowledge several important limitations in our analysis. First and foremost, due to the lack of data on contact patterns, we elaborated and simulated five illustrative scenarios to parameterize our model, as there were no available data (excluding patterns by income decile from Mexico; scenario 4) for deriving income-specific transmission rates. Future research should therefore aim to roll out individual contact surveys, across socioeconomic groups in low- and middle-income countries including Ethiopia, similar to those presented by Mossong et al. in the POLYMOD study and in related approaches designed by Kiti et al. applied to Kenya [31], [32]. In Mossong et al, for example, participants were randomly allocated a day to document information on demographic characteristics (age and sex) of all individuals with whom they had contact, and the venue, duration and nature of each interaction in a “contact diary” [31]. For the surveys we would propose, participants would be asked to additionally note the general SES category of the individual with whom they interact, using occupation as a potential proxy. Nonetheless, while making strong assumptions for the relative magnitude of contact among quintiles and the effects of social mixing in shaping differences in the risk of transmission across quintiles, our study could still provide reasonable preliminary intuitions, including conservative uncertainty intervals, that can prompt future extensions to empirically-derived income-stratified modeling approaches and highlight the specific needs for data collection.

Another limitation is the lack of data for select parameters, for which we relied on the published literature and approximate data sources and imputations. For instance, we assumed that crude birth rates by quintile were proportional to total fertility rates by quintile to estimate quintile-specific crude birth rates. Additionally, we made a number of assumptions to grossly approximate a CFR gradient. Future work could for example utilize individual-level survey data (e.g. from DHS) to directly estimate crude birth rates and CFRs by SES [33], [34].

In addition, our model did not consider heterogeneities beyond income, notably age, which is a critical determinant of measles transmission [35]. However, without available disaggregated data by both age and income, additionally stratifying our model by age would further complicate our analysis without providing additional insight to our main findings. While younger age groups (e.g. schoolchildren [35]) may selectively contact one another and chiefly contribute to overall transmission, there are likely additional heterogeneities in mixing, infection (and in treatment access) among children from different income groups. Consequently, while we may overlook transmission processes and mortality by failing to account for differential mixing within certain age groups, we would still expect to observe potentially similar income-associated gradients. Furthermore, the chief intent of our paper is to provide a first attempt at explicitly incorporating equity in measles modeling and laying out a tentative distributional impact framework from which to build upon more precisely in the future.

Finally, we considered stylized cases of vaccination, assuming “ex-ante” a certain coverage of the population. More thorough modeling of routine immunization strategies as well as future extensions to pulse vaccinations and supplementary immunization activities delivered at varying time points would likely alter the dynamics we observe across income groups.

In summary, mechanistic models of infectious diseases stratified by socioeconomic drivers can be instrumental for grounding biological and ecological understanding of disease, and the populations they affect, within the broader social and political contexts they inhabit. As disease dynamics are driven by biological determinants and shaped by social disparities, this framework may be a steppingstone towards defining a more accurate portrait of distributional disease burden. Further research focusing on other non-biological risk groups, such as immigrant communities, indigenous groups, and other disadvantaged populations, can help inform a more expanded understanding of the emergence of disease outbreaks. Simple models such as the one we propose here, when calibrated with empirical data, can be useful for diseases where social factors like income doubly affect vulnerabilities in contracting diseases and in accessing available health care. Distributional predictions may better inform which interventions to support, such as nutritional supplements, and which populations to focus on when expanding existing interventions, such as vaccination, with the ultimate aim of achieving greater health equity.

## Declaration of Competing Interest

The authors declare that they have no known competing financial interests or personal relationships that could have appeared to influence the work reported in this paper.

## Footnotes

1 Note also that demographic data (e.g. population sizes and birth rates) is not available as disaggregated across both age groups and SES; and, little evidence is available that supports age group variations in mixing across SES groups. Therefore, our SIR model was not stratified by age, but solely by SES, in keeping with the prime purpose of our study.

2 Under scenario 1, the corresponding R_11_ and $\sum_{j=2}^{5}R_{1j}$ terms would then each equal 0.5*17.9. Under scenario 2, R_11_ = 0.9*17.9 and $\sum_{j=2}^{5}R_{1j}=0.1*17.9$. Under scenario 3, R_11_ = 0.1*17.9 and $\sum_{j=2}^{5}R_{1j}=0.9*17.9$.
