# Supplementary material for: Incorporating equity in infectious disease modeling: Case study of a distributional impact framework for measles transmission
Source: Vaccine. 2021 May 18;39(21):2894–900. doi: 10.1016/j.vaccine.2021.03.023 (PMC8117973; doi:10.1016/j.vaccine.2021.03.023)
Supplement: Supplementary data 1 [file mmc1.pdf]

Supplementary webappendix

1. Derivation of crude birth rates and case fatality ratios

Crude birth rates

Data on crude birth rates (CBRs) and total fertility rates (TFRs) by wealth quintile from the 2016 Ethiopian Demographic and Health Survey (DHS) were used to derive quintile-specific CBRs [1]. The total (population-level) CBR was assumed to be the average of the CBR values by quintile. The ratio of CBRs by quintile was assumed to equal the ratio of TFRs by quintile: the ratio of CBRs for each pair of quintiles was assumed to be proportional to the ratio of TFRs for each pair of quintiles. Under this assumption, the CBR for quintile *j* could be rewritten as the product of the CBR of the first quintile and the ratio of TFR for quintile *j* and the TFR for the first quintile (see equations S1-S3 below). The procedure is captured in the following equations:

$$\frac{TFR_j}{TFR_1} = \frac{CBR_j}{CBR_1} \quad , \qquad \text{(S.1)}$$

$$\frac{5CBR_{tot}}{(1+\frac{TFR_2}{TFR_1}+\frac{TFR_3}{TFR_1}+\frac{TFR_4}{TFR_1}+\frac{TFR_5}{TFR_1})} = CBR_1 \ , \qquad \text{(S.2)}$$

$$CBR_j = \frac{TFR_j}{TFR_1} * CBR_1 \ . \qquad \text{(S.3)}$$

Case-fatality ratios (CFRs)

We drew from estimated CFRs (year 2015) from Portnoy and colleagues [2]: specifically, we used the estimated median overall CFR value for the low-income country category, i.e. 1.6% (0.5 to 5.0%). As an approximation, this CFR was subsequently distributed across each income

quintile, using the relative distribution (across wealth quintiles) in the percentage among children for whom treatment was sought from the Ethiopian DHS [1], as an attempt to capture differences in healthcare access across different socioeconomic groups in the Ethiopian population. The following proxied CFR estimates (from quintile 1 to 5) were then derived: 2.18%; 1.89%; 1.60%; 1.31%; and 1.02%.

2. Derivation of transmission rate matrices

Simulating the transmission matrices for scenarios 1, 2, and 3

Below we describe our methods for generating each simulated transmission matrix for scenarios 1, 2, and 3 described in the main text (Methods section). For simplicity, note that we assumed symmetry in the transmission matrices: $\beta_{ij} = \beta_{ji}$.

1. Draw 5 values of $R_{0,i}$ from a uniform distribution with defined minimum (10) and maximum (22) values that yields a mean value of 16 (see Table 1).

2. Define all R_{ii} values (diagonal terms) as the ordered sequence of $0.5*R_{0,i}$ (scenario 1), $0.9*R_{0,i}$ (scenario 2), and $0.1*R_{0,i}$ (scenario 3).

3. Define the sum of all R_{ij} values ($i \neq j$, off-diagonal terms) as $0.5*R_{0,i}$ (scenario 1), $0.1*R_{0,i}$ (scenario 2), and $0.9*R_{0,i}$ (scenario 3).

4. Sample 4 values for the R_{1j} terms (for quintile 1, $j \neq 1$) from a uniform distribution with mean equal to the previously sampled sum of the 4 R_{1j} terms (in step 3) divided by 4. Repeat this step *n*=100 times.

5. Extract the sums of each of the *n* combinations of R_{1j} terms from step 4, and select those combinations that yield a sum that equals the previously sampled sum of R_{1j} terms in step 3 (rounded to the nearest tenth digit).

6. Identify the combination which yields the sum that most closely approximates the (unrounded) previously sampled sum of R_{1j} terms from step 4 to select the optimal parameters for the R_{1j} terms (i.e. $R_{1,2}$, $R_{1,3}$, $R_{1,4}$, $R_{1,5}$).

7. Repeat steps 4-6 for all R_{ij} terms (i≠1; for quintiles i=2; 3; 4; 5), sampling only 3 values (for matrix row i=2), 2 values (for matrix row i=3), and 1 value (for matrix row i=4), respectively, for each subsequent quintile, given that matrix symmetry is assumed.

We repeat steps 1-7 above for each of the *n*=1000 SIR model iterations and translate the simulated matrix into β_{ij} (in the SIR model equations (1) in the main text) terms, using the following relationship:

$$\beta_{ij} = \frac{R_{ij} * \gamma}{N_{i,0}} , \qquad \text{(S.4)}$$

where $N_{i,0}$ represents the initial population size of quintile i ($N_{i,0} = N_0$) and γ is the recovery rate.

Scenario 4 using Mexican "contact" survey

We also derived contact matrices and corresponding transmission matrices from the Leo et al. 2016 study [3]. The ratio "number of links" (phone calls or texts) observed between individuals in all possible decile pairings and the number of such links expected in a social network with random mixing, or the $L(s_i,s_j)$ terms, were directly extracted from the study (Figure 3b, in Leo et al. [3]). The values for the natural log of $L(s_i,s_j)$ for each of the fifteen possible (i, j) quintile combinations were extracted from the figure. The population-wide transmission rate β was set to equal the weighted average of the contact rates between all quintiles i and j. All cross-quintile contact rates c_{ij} were rewritten in terms of the within-quintile contact rate for the first quintile (c_{11}) by equating them to the product of c_{11} and the ratio of L_{ij} to L_{11}. We translated cross-decile L_{ij} terms to cross-quintile terms assuming that: the first quintile would correspond to the first

decile, the second quintile to the third decile, the third quintile to the fifth decile, the fourth quintile to the seventh decile, and the fifth quintile to the ninth decile. These c_{ij} terms were included in the equation for β, which was rearranged to isolate c_{11}. All other contact rates were then calculated using the aforementioned relationship between c_{ij}, c_{11}, and L_{ij}/L_{11}. Finally, β_{ij} transmission rates were set to equal the product of the probability of successful transmission to quintile j (denoted f) and c_{ij} to yield transmission matrices. Below we report the formulas used:

$$c_{12} = c_{11}\frac{L_{12}}{L_{11}} = c_{11}\frac{\overline{L_{13}}}{\overline{L_{11}}} \qquad ,$$

$$c_{13} = c_{11}\frac{L_{13}}{L_{11}} = c_{11}\frac{\overline{L_{15}}}{\overline{L_{11}}} \qquad ,$$

$$c_{14} = c_{11}\frac{L_{14}}{L_{11}} = c_{11}\frac{\overline{L_{17}}}{\overline{L_{11}}} \qquad ,$$

$$c_{15} = c_{11}\frac{L_{15}}{L_{11}} = c_{11}\frac{\overline{L_{19}}}{\overline{L_{11}}} \qquad ,$$

$$c_{22} = c_{11}\frac{L_{22}}{L_{11}} = c_{11}\frac{\overline{L_{33}}}{\overline{L_{11}}} \qquad ,$$

$$c_{23} = c_{11}\frac{L_{23}}{L_{11}} = c_{11}\frac{\overline{L_{35}}}{\overline{L_{11}}} \qquad ,$$

$$c_{24} = c_{11}\frac{L_{24}}{L_{11}} = c_{11}\frac{\overline{L_{37}}}{\overline{L_{11}}} \qquad ,$$

$$c_{25} = c_{11}\frac{L_{25}}{L_{11}} = c_{11}\frac{\overline{L_{39}}}{\overline{L_{11}}} \qquad ,$$

$$c_{33} = c_{11}\frac{L_{33}}{L_{11}} = c_{11}\frac{\overline{L_{55}}}{\overline{L_{11}}} \qquad ,$$

$$c_{34} = c_{11}\frac{L_{34}}{L_{11}} = c_{11}\frac{\overline{L_{57}}}{\overline{L_{11}}} \qquad ,$$

$$c_{35} = c_{11}\frac{L_{35}}{L_{11}} = c_{11}\frac{\overline{L_{59}}}{\overline{L_{11}}} \qquad ,$$

$$c_{44} = c_{11}\frac{L_{44}}{L_{11}} = c_{11}\frac{\overline{L_{77}}}{\overline{L_{11}}} \qquad ,$$

$c_{45} = c_{11}\frac{L_{45}}{L_{11}} = c_{11}\frac{\overline{L_{79}}}{\overline{L_{11}}}$,

$c_{55} = c_{11}\frac{L_{55}}{L_{11}} = c_{11}\frac{\overline{L_{99}}}{\overline{L_{11}}}$,

$$c_{11} = \frac{\beta*(N_1^2+N_2^2+N_3^2+N_4^2+N_5^2+2N_1N_2+2N_1N_3+2N_1N_4+2N_1N_5+2N_2N_3+2N_2N_4+2N_3N_4+2N_3N_5+2N_4N_5)}{N_1^2f_1+N_2^2f_2\frac{L_{22}}{L_{11}}+N_3^2f_3\frac{L_{33}}{L_{11}}+N_4^2f_4\frac{L_{44}}{L_{11}}+N_5^2f_5\frac{L_{55}}{L_{11}}+N_1N_2(f_1+f_2)\frac{L_{12}}{L_{11}}+N_1N_3(f_1+f_3)\frac{L_{13}}{L_{11}}+N_1N_4(f_1+f_4)\frac{L_{14}}{L_{11}}+N_1N_5(f_1+f_5)\frac{L_{15}}{L_{11}}+N_2N_3(f_2+f_3)\frac{L_{23}}{L_{11}}+N_2N_4(f_2+f_4)\frac{L_{24}}{L_{11}}+N_2N_5(f_2+f_5)\frac{L_{25}}{L_{11}}+N_3N_4(f_3+f_4)\frac{L_{34}}{L_{11}}+N_3N_5(f_3+f_5)\frac{L_{35}}{L_{11}}+N_4N_5(f_4+f_5)\frac{L_{45}}{L_{11}}}$$

. (S.5)

L_{ij} indicates the ratio of observed and expected links for quintiles *i* and *j* (as described previously); $\overline{L_{ij}}$ indicates the ratio of observed and expected links for quintiles *i* and *j* for the corresponding deciles reported in Leo et al.; N_i is the number of individuals in quintile *i* and f_i=f=constant is the probability of successful infection for individuals in quintile *i*.

Scenario 5 assuming homogeneous mixing

We assumed a common R_0 value across all quintiles (drawn from a uniform distribution with mean 16). As a result, $\beta_{ij} = \frac{R_0*\gamma}{N_{total}}$, where N_{total} represents the total population size.

For all five scenarios (1, 2, 3, 4, and 5), the susceptible S and recovered R populations' size for quintiles 1 to 5 were initialized at the number of unvaccinated and successfully vaccinated individuals, respectively, among 15,000 individuals per quintile (reflective of the population sizes for each wealth quintile as reported in the DHS using quintile-specific vaccine coverage levels) [1]. We initialized infection by seeding one infected case in each quintile and assumed that no individuals were deceased at the start of the simulation.

3. **Disparities and estimation of concentration indices**

To estimate the concentration index (CI) resulting from each distributional vaccination case (see Methods section in the main text), we calculated the cumulative sum of each quintile's proportional population size (such that the cumulative sum at quintile 5 equals 1), and the cumulative sum of each quintile's proportional number of measles deaths. The ensuing concentration curve is then constructed as a plot of the cumulative share of number of deaths for each quintile against their cumulative share of the total population. Subsequently, the CI is computed as twice the area under the concentration curve above the "line of equality" [4].

4. Supplemental figures

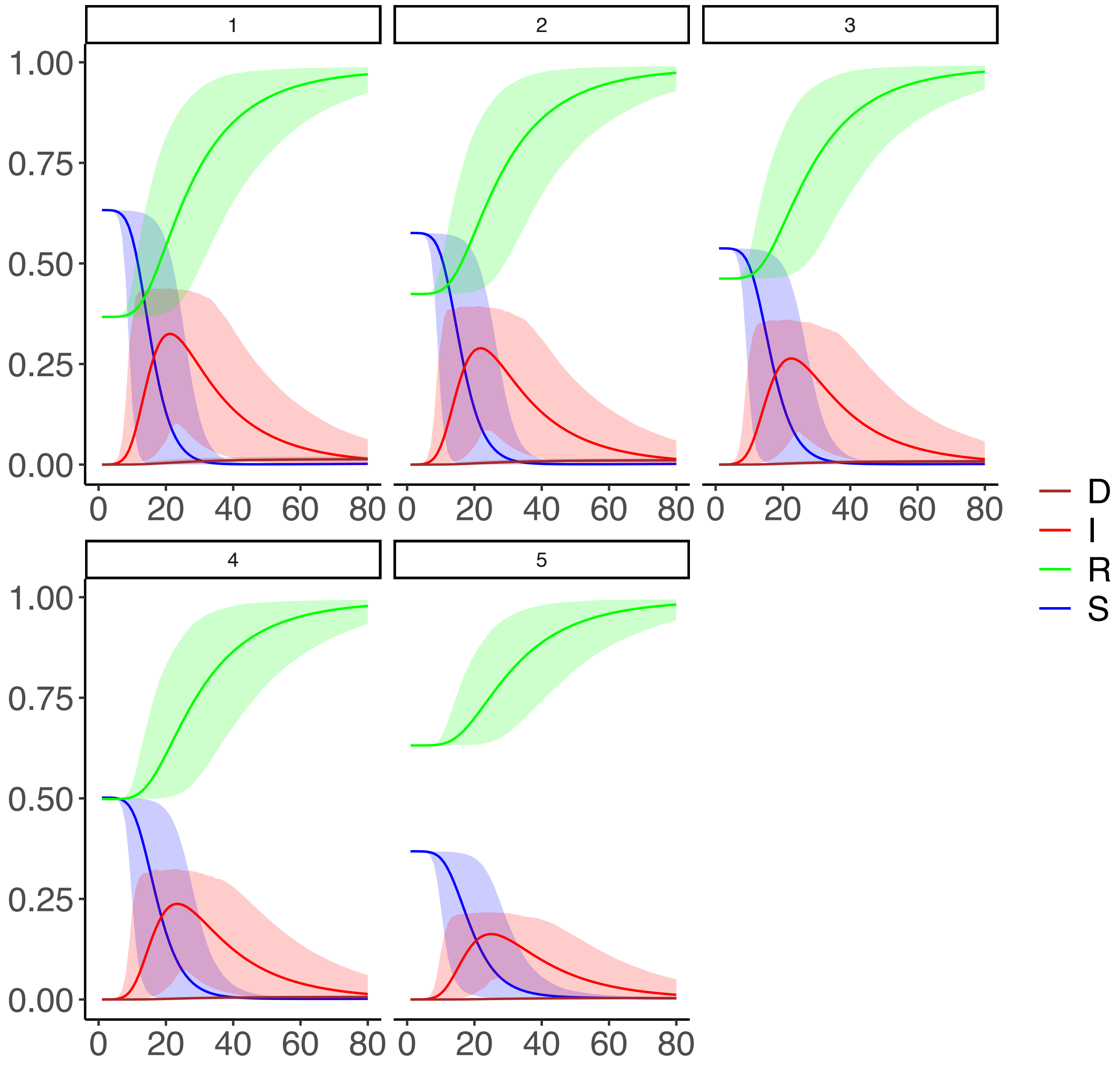

Figure S1: Susceptible (blue), Infected (red), Recovered (green), and Deceased (brown) model dynamics for all quintiles (1=poorest; 5=richest) under transmission scenario 1. Solid lines indicate mean values while shaded areas indicate 95% uncertainty intervals for each compartment.

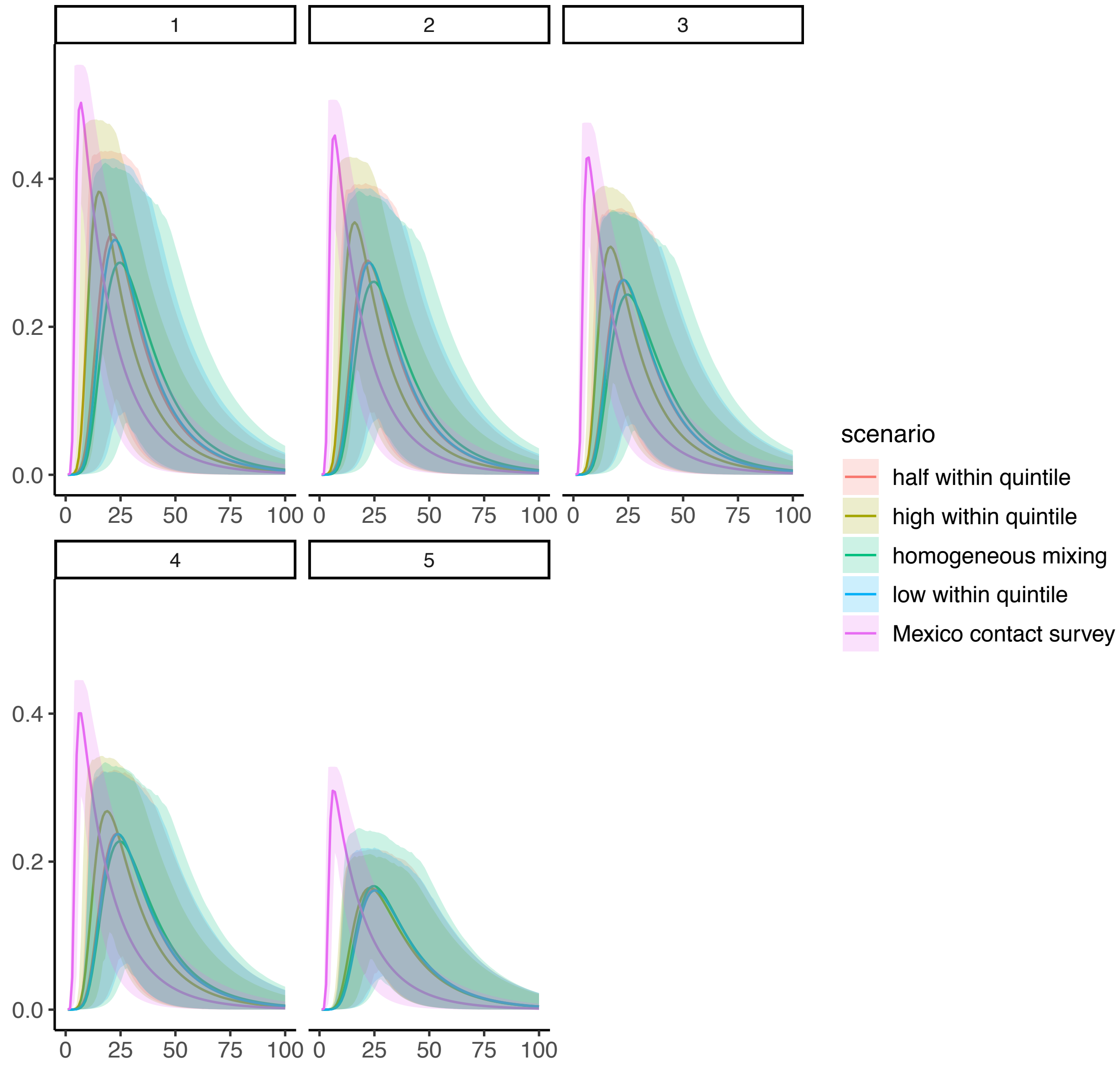

Figure S2: Model dynamics of the infected (I) population (proportion) for transmission scenarios 1 to 5, by income quintile (1=poorest; 5=richest). Solid lines indicate mean values while shaded areas indicate 95% uncertainty intervals for each scenario.

Webappendix references

1. Central Statistical Agency, The DHS Program - ICF. Ethiopia Demographic and Health Survey 2016 [Internet]. 2017. Available from: https://dhsprogram.com/pubs/pdf/FR328/FR328.pdf

2. Portnoy A, Jit M, Ferrari M, Hanson M, Brenzel L, Verguet S. Estimates of case-fatality ratios of measles in low-income and middle-income countries: a systematic review and modelling analysis. Lancet Global Health. 2019 Apr;7(4):e472–81

3. Leo Y, Fleury E, Alvarez-Hamelin JI, Sarraute C, Karsai M. Socioeconomic correlations and stratification in social-communication networks. J R Soc Interface. 2016 Dec 31;13(125):20160598.

4. O'Donnell O, van Doorslaer E, Wagstaff A, Lindelow M. Analyzing Health Equity Using Household Survey Data : A Guide to Techniques and Their Implementation [Internet]. Washington, DC: The International Bank for Reconstruction and Development / The World Bank; 2008. 220 p. Available from: https://openknowledge.worldbank.org/bitstream/handle/10986/6896/424800ISBN978011OFFICIAL0USE0ONLY10.pdf?sequence=1&isAllowed=y
